# Supplementary material for: Chromothripsis during telomere crisis is independent of NHEJ, and consistent with a replicative origin
Source: Genome Res. 2019 May;29(5):737–49. doi: 10.1101/gr.240705.118 (PMC6499312; doi:10.1101/gr.240705.118)
Supplement: Supplemental Material [file supp_gr.240705.118_Supplemental_file_1.zip › contigs/annotated_contigs/DB51/contig.2.DB51_length_784_mean_cov_9.33035714286.docx]

**DB51_length_784_mean_cov_9.33035714286**

```
CATCAATATGATAGTGGCCTTTCTGAAGTAGAAATTATTACCATTCCTACAAATGATGGATCCATCGTGGTTGGAATAGTCTTGGAAAT
 >chr7:143161728-143162131 + E=1e-227
TATAAACATAACTGGTAGGTCTAGAGTTGGCAAATGTATGACTATAAAATAAACTGTCAGTAACTATTAATGCCCAAATTATAATGGCT

AATGCAGCCATCCTTAATTTCTGCTCCTCAGTGCTCCTGCCCTCTCTCCTCTGTAAACTATTTCCCCAGTTACTTTTGTAATGGTGGTG

GGTAGAAGTGGTTCCAATCATCTATATTTCATGTTTTCATGCAAAGGGTACCTTATGGCATATTCTTCCTCTCCACGTCTGTCTCTCTG

GTTGATATCCATGTCCATGAGCTCCCTAAAGGGGAGCTGTCCCT|ATG|TTTCCCTCTCCTAGGTCCAGCAGGATAGTCTGGACAGGGA
                                             >chr7:143157440-143157824 + E=3e-219
TAGAATCTGAATATTTTATTTCTGGCAAAATCTTTATGTCTTTGCTCGTTGTTTTCCTATCTATGTCATGACAACTCTTGTAAACATTC

CATTTTTTTCTGTTCAAGGCAGAAATCATAGGCCAGTCCTATCAAAGGTTCTCAGTCAATACTATTCTGCATTATAGCATTATCTTCCA

AGGAGCATGCTTTCAATATATTGACTTTTTACATTTACTATTTTTAACTACTATGGACAAATTTTATATTATGTAAGACTGTCTCAGGC

ATGATATTCGTTGATTCTTAGTCACTTTGGGGAAATTTATTATAACCATTTTATTTTGCATAGTTTTGCATAGT
```
